# Yeast functional screen to identify genes conferring salt stress tolerance in *Salicornia europaea*

**DOI:** 10.3389/fpls.2015.00920

**Published:** 2015-10-28

**Authors:** Yoshiki Nakahara, Shogo Sawabe, Kenta Kainuma, Maki Katsuhara, Mineo Shibasaka, Masanori Suzuki, Kosuke Yamamoto, Suguru Oguri, Hikaru Sakamoto

**Affiliations:** ^1^Institute of Plant Science and Resources, Okayama UniversityKurashiki, Japan; ^2^Graduate School of Biological Sciences, Nara Institute of Science and TechnologyIkoma, Japan; ^3^Faculty of Bioindustry, Tokyo University of AgricultureAbashiri, Japan; ^4^Advanced Life Science Institute Inc.Wako, Japan

**Keywords:** *Salicornia europaea* L., yeast functional screen, salt stress, short peptide, thaumatin-like protein, coiled-coil protein

## Abstract

Salinity is a critical environmental factor that adversely affects crop productivity. Halophytes have evolved various mechanisms to adapt to saline environments. *Salicornia europaea* L. is one of the most salt-tolerant plant species. It does not have special salt-secreting structures like a salt gland or salt bladder, and is therefore a good model for studying the common mechanisms underlying plant salt tolerance. To identify candidate genes encoding key proteins in the mediation of salt tolerance in *S. europaea*, we performed a functional screen of a cDNA library in yeast. The library was screened for genes that allowed the yeast to grow in the presence of 1.3 M NaCl. We obtained three full-length *S. europaea* genes that confer salt tolerance. The genes are predicted to encode (1) a novel protein highly homologous to thaumatin-like proteins, (2) a novel coiled-coil protein of unknown function, and (3) a novel short peptide of 32 residues. Exogenous application of a synthetic peptide corresponding to the 32 residues improved salt tolerance of *Arabidopsis*. The approach described in this report provides a rapid assay system for large-scale screening of *S. europaea* genes involved in salt stress tolerance and supports the identification of genes responsible for such mechanisms. These genes may be useful candidates for improving crop salt tolerance by genetic transformation.

## Introduction

Soil salinity is one of the most critical environmental factors limiting the productivity of agricultural crops, with adverse effects on germination, plant growth and crop yield. The problem has been aggravated by agricultural practices such as irrigation. Today, worldwide 20% of total cultivated lands and 33% of irrigated agricultural lands are aﬄicted by high salinity. Furthermore, the salinized areas are increasing at a rate of 10% annually for various reasons, including low precipitation, high surface evaporation, weathering of native rocks, irrigation with saline water, and poor cultural practices. It has been estimated that more than 50% of the arable land would be salinized by the year 2050 (Jamil et al., 2011).

Generally, salt stress causes osmotic stress, ionic stress such as toxicity of Na^+^, and oxidative stress by reactive oxygen species (ROS) in plants. These stresses result in water loss, nutritional disorders, alteration of metabolic processes, membrane disorganization, reduction of cell division and expansion, and genotoxicity. Together, these effects reduce plant growth, development, and survival (Carillo et al., 2011).

Because of their immobile nature, plants have evolved mechanisms to sense and respond to salt stress (Katsuhara et al., 2003; Verslues et al., 2006). For avoidance of dehydration, osmoprotectant synthesis, cell wall adjustment (hardening or softening) and regulation of aquaporin expression are carried out. For avoidance of ionic toxicity, toxic ions are exported from cells or compartmentalized into vacuoles through membrane-localized transporters. For cell protection from oxidative stress, plants have various ROS scavengers. Nevertheless, glycophytes including most of crops cannot withstand high salinity.

Halophyte plants growing near seashores have adapted to soil salinity. Identification of genes responsible for salt tolerance in halophyte plants is important for agricultural development since transformation of such genes into crops may lead to improved productivity of crops under salt stress conditions. Many halophytes have special salt-secreting structures like a salt gland or salt bladder. Genes involved in these structures cannot be used for generation of transgenic salt-tolerant crops, since most of glycophytes does not have such structures.

*Salicornia europaea* L. is one of the most highly salt-tolerant species. It can withstand extreme saline environments of more than 1 M NaCl and shows optimal growth under moderate NaCl concentrations (200 mM) (Wang et al., 2009; Ma et al., 2013). It does not have special salt-secreting structures, and is thus good model species for studying the common mechanisms underlying plant salt tolerance (Shi et al., 2006). *S. europaea* accumulates Na^+^ in shoot vacuoles (Lv et al., 2012), thus reducing the toxicity of Na^+^ in the cytoplasm and limiting the osmotic potential of vacuoles to preserve cellular turgor pressure and expansion under high salt conditions. Coincidentally, glycine betaine, a major osmoprotectant, is also increased in *S. europaea* leaves by salinity, resulting in a decrease in osmotic potential (Moghaieb et al., 2004). ROS are generated in plants under high salt conditions and damage multiple cellular components. To alleviate oxidative damage, *S. europaea* utilizes ROS scavengers such as superoxide dismutase, catalase, and carotenoid (Aghaleh et al., 2011; Chen et al., 2011). Recently, *SeNHX1* (vacuolar Na^+^/H^+^ antiporter), *SeCMO* (glycine betaine biosynthesis), *SePSY* (carotenoid biosynthesis), and *SeLCY* (carotenoid biosynthesis) were isolated from *S. europaea* (Han et al., 2008; Zhou et al., 2008; Wu et al., 2010; Chen et al., 2011; Zhang et al., 2014). The expression of these genes was correlated with salt concentration. Constitutive expression of these genes improves salt tolerance in transgenic *Arabidopsis*, tobacco, and alfalfa. Glycophytes also carry homologs of these genes. For example, *Arabidopsis LCY* improves salt tolerance in transgenic tobacco, similar to *SeLCY*. There are no significant differences in the extent of salt tolerance between *SeLCY*- and *Arabidopsis LCY*-transformed plants (Chen et al., 2011). *S. europaea* has probably evolved mechanisms for salt tolerance that are common to glycophytes.

To identify genes involved in salt tolerance in *S. europaea*, proteome and transcriptome approaches have been employed (Wang et al., 2009; Ma et al., 2013). As another powerful approach to identifying genes involved in salt tolerance, functional screening of cDNA libraries from halophytes in microorganisms has been reported (Eswaran et al., 2010). By using this approach, Yamada et al. (2010) identified the *S. europaea* gene *SeFLA* encoding an arabinogalactan protein that enhances salt tolerance in *Escherichia coli*.

Here, to identify candidate genes encoding key proteins involved in salt tolerance in *S. europaea*, we performed functional screening of an *S. europaea* cDNA library in a yeast expression vector. The library was transformed into yeast and screened for genes that enabled growth in the presence of high-concentration NaCl. This approach identified three candidate genes (*SeNN8*, *24*, and *43*).

## Materials and Methods

### Plant Materials

The seeds of *S. europaea* L. were harvested around Lake Notoro in the eastern part of Hokkaido. Methods of cultivation and salt treatment of *S. europaea* plants were as reported elsewhere (Momonoki et al., 2009). Three-month-old plants were used to construct a cDNA library. *Arabidopsis thaliana* Col-0 plants were routinely grown at 22°C under continuous white light on solid MS media (Murashige and Skoog, 1962) containing 1% w/v sucrose and 0.5% w/v gellan gum. Seedlings were used for salt tolerance assays 7 days after germination.

### Construction of the *S. europaea* cDNA Library

Total RNA was isolated from 3-month-old *S. europaea* plants using an RNeasy Plant Mini kit (Qiagen, Germany) according to manufacturer instructions. After DNase treatment and phenol–chloroform extraction, the RNA was reverse-transcribed to cDNA using an In-Fusion SMARTer^TM^ Directional cDNA Library Construction Kit (Clontech, USA). Vector DNA was PCR-amplified by using a yeast expression vector pYES2.1/V5-His/lacZ (Invitrogen, USA) as a template and two oligonucleotide primers (5′-GTACTCTGCGTTGATACCACTGCTTGGATCCAAGGGCGAGCTTA-3′ and 5′-TGGTGTCTTATCGTACCCCGTCTAGAGGGCCGCATCATG-3′). After *Dpn*I treatment to degrade the template plasmid, the PCR-amplified vector DNA was purified using Nucleo Spin Gel and PCR Clean-up kit (MACHEREY-NAGEL, Germany). The *S. europaea* cDNA was cloned into the PCR-amplified vector DNA by In-fusion reaction. In the resulting plasmids, *S. europaea* cDNAs were inserted into the *Bam*HI-*Xba*I site of pYES2.1/V5-His/lacZ. The plasmid libraries were transformed into *E. coli* HST08 (Takara, Japan) and 233,760 independent clones were obtained. To confirm the existence of various genes in the constructed library, arbitrary six plasmids were isolated from *E. coli* and digested with *BamHI* and *XbaI*.

### Yeast Functional Screening

The library plasmids were extracted from *E. coli* and transformed into *Saccharomyces cerevisiae* BY4741 using PEG-lithium acetate-based transformation protocols (Eswaran et al., 2010). Yeast colonies harboring *S. europaea* cDNA library plasmids were selected on synthetic complete media containing 2% glucose and lacking uracil. To screen salt-tolerant yeast colonies, the resulting transformants were washed with liquid synthetic complete media containing 2% galactose and 1% raffinose, lacking uracil (SGR-Ura), and then plated on SGR-Ura solid media containing 1.3 M NaCl. Primary salt-tolerant colonies were harvested and re-plated on SGR-Ura solid media containing 1.6 M NaCl. The plasmids in salt-tolerant colonies were rescued and re-transformed into *S. cerevisiae* BY4741. The improved salt tolerance in resulting transformants was confirmed by adjusting overnight SGR-Ura cultures of these colonies to *OD*_600_ = 0.5 and spotting serial dilutions (1, 1:10, and 1:100) onto SGR-Ura solid media containing NaCl, LiCl, or H_2_O_2_. *S. europaea* cDNAs harbored in the salt-tolerant colonies were sequenced.

### Plant Salt Tolerance Assay

Seven-day-old *Arabidopsis* seedlings were transferred onto filter papers soaked with a SeNN43 synthetic peptide solution (5 μM, NH_2_-MFALLTTAGVSAHTEKLTDINGKYSFHSNTLT-COOH, Eurofins Genomics), a control peptide solution (5 μM, NH_2_-TEFVKGYSLTNMNLKTFSTGHALDTAIHLATS-COOH, Eurofins Genomics), or water. After 24 h, the seedlings were transferred onto MS solid media containing 100 or 200 mM NaCl. For survival rate analysis, the numbers of plants with chlorotic cotyledons on media containing NaCl were scored for 7 days. For ion leakage measurements and staining by propidium iodide (PI), seedlings were used after 4 days growth on media containing NaCl. Ion leakage from leaves was measured as described previously (Nanjo et al., 1999). For PI staining, roots were vacuumed in 10 μg/ml PI solution for 1 min, three times, and washed with water. PI fluorescence was monitored by confocal scanning-laser microscopy TCS-SP5 (Leica Microsystems, Germany). For shoot fresh weights measurements, seedlings were grown for 4 days on media containing NaCl and transferred onto normal MS media for an additional 3 days. For root elongation measurements, seedlings pretreated with the peptides were transferred onto vertical MS plates containing 100 mM NaCl. Increases in root length were measured for 7 days. Statistical significance was determined by Steel-Dwass test (*P* < 0.05).

### Subcellular Localization Analysis

To construct *SeNN8-green fluorescent protein (GFP)* and *SeNN24-GFP* fusion genes, open reading frame sequences of *SeNN8* and *SeNN24* were PCR-amplified by using plasmids isolated from salt-tolerant yeasts #8 and #24 as a template and two sets of oligonucleotide primers (for *SeNN8*: 5′- CACCATGACAGAGGAAGAAGTGG-3′ and 5′- CAATTCTCCCTTACTAGCATC-3′, for *SeNN24*; 5′-CACCATGATGAGGAATTTATTATTATTTTCC-3′ and 5′-GGACTTAGGAGGGCAGAA-3′). The PCR-amplified fragments were subcloned into pENTR/D-TOPO vector^TM^ (Invitrogen) by TOPO cloning methods, followed by integration into the destination vector pGWB5 (Nakagawa et al., 2007) using Gateway LR clonase (Invitrogen), respectively. The resulting plasmids were introduced into onion epidermal cell layers by using particle bombardment (IDERA GIE-III, Tanaka, Japan) as described previously (Chiu et al., 1996). GFP fluorescence was monitored by confocal scanning-laser microscopy TCS-SP5 (Leica Microsystems, Germany). For cell plasmolysis, the onion epidermal strips were exposed to 1 M sucrose.

To investigate the subcellular localization of SeNN43, a fluorescein isothiocyanate (FITC)-conjugated SeNN43 synthetic peptide (Biologica) was used. The FITC-conjugated peptide (50 μM) was provided to *Arabidopsis* for 5 h. After three times washing, FITC fluorescence was monitored by using TCS-SP5.

### PCR Analysis

To demonstrate the existence and expression of *SeNN8*, *24*, and *43* in *S. europaea*, genomic PCR and RT-PCR were performed. Seeds of *S. europaea* are sterilized and sown on solid MS media (Murashige and Skoog, 1962) containing 3% w/v sucrose and 0.8% w/v agar and then routinely grown at 25°C under continuous white light. Two-weeks-old seedlings were used for genomic DNA and RNA extraction. Genomic DNA was isolated using a DNeasy Plant Mini kit (Qiagen, Germany) according to manufacturer instructions. Total RNA was isolated as described above. The RNA was reverse-transcribed to cDNA using a Reverscript I (Wako, Japan). Deduced open reading frame sequences of *SeNN8*, *24*, and *43* were PCR-amplified by using genomic DNA and cDNA as templates and three sets of oligonucleotide primers (for *SeNN8* and *SeNN24*: the same primer sets described above, for *SeNN43*: 5′-CACCATGTTTGCTCTGCTGACG-3′ and 5′-AGTGAGAGTATTAGAATGAAAG-3′). The amplified fragments by genomic PCR were sequenced.

### Apoptosis-like Programmed Cell Death (AL-PCD) Assay

Root hairs with AL-PCD were scored as described previously (Hogg et al., 2011). *Arabidopsis* roots were treated with water, the control peptide (5 μM), or the SeNN43 peptide (5 μM) for 24 h as described above. After three times washing, the roots were stained with a 1 μg/ml fluorescein diacetate (FDA) and immediately observed under white light and fluorescent light using TCS-SP5. Three types of root hairs were scored: FDA-stained root hairs as living cells, no-stained root hairs with retraction of the cytoplasm as cells undergoing AL-PCD, and root hairs showing neither FDA staining nor retraction of the cytoplasm as necrotic cells. The percentage for each category was calculated as a percentage of the total number of roots hairs scored (typically ∼300). Statistical significance was determined by student *t*-test (*P* < 0.05).

## Results

### Functional Screening for Genes Involved in Salt Tolerance in *S. europaea*

**FIGURE 1:**
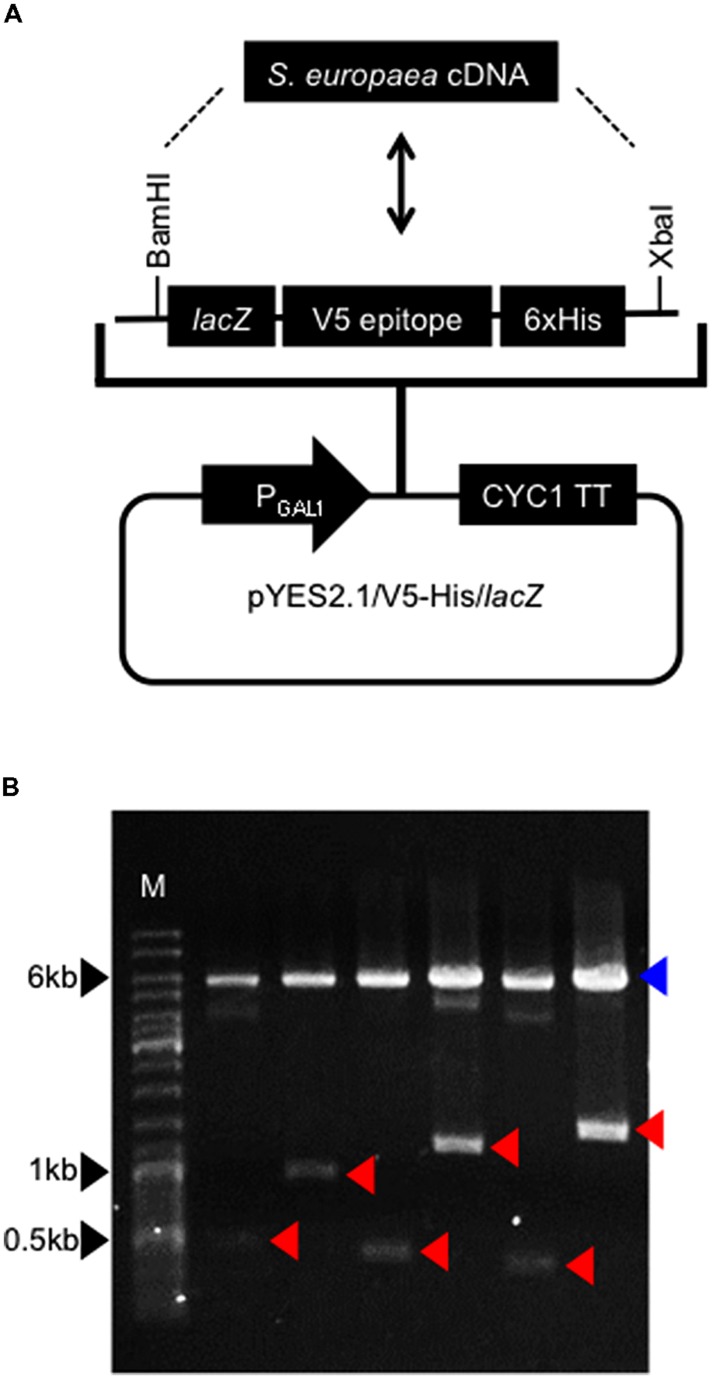
**Construction of *Salicornia europaea* cDNA library. (A)** Structure of a yeast expression vector. *S. europaea* cDNAs were introduced into *BamHI*-*XbaI* site of the vector. P_GAL1_ and CYC1TT indicate *GAL1* promoter and *CYC1* transcription termination signal, respectively. In resulting plasmids, *lacZ*, V5 epitope and 6xHis sequences were eliminated. **(B)** Insert lengths in constructed library. Arbitrary six plasmids were digested with *BamHI* and *XbaI*. Red arrowheads indicate bands of inserts. A blue arrowhead indicates a band of vector. M indicates a DNA size marker.

To identify genes involved in salt tolerance in *S. europaea* at the mature stage, we performed functional screening of its cDNA library in a yeast expression vector. Total RNAs were isolated from 3-month-old plants grown under 3% NaCl conditions, and reverse transcribed using In-Fusion SMARTer^TM^ Directional cDNA Library Construction Kit. The cDNAs were inserted into the *BamHI*/*XbaI* site of a yeast expression vector pYES2.1/V5-His/*lacZ* (**Figure 1A**). In resulting plasmids, *S. europaea* genes were located downstream of galactose-inducible GAL1 promoter, with 5′ to 3′ direction. The plasmid libraries were transformed into *E. coli* and 233,760 independent clones were obtained. To confirm the existence of various genes in resulting library, arbitrary six plasmids were digested with *BamHI* and *XbaI* (**Figure 1B**). The lengths of digested fragments correspond to those of *S. europaea* genes unless the genes have *BamHI* or *XbaI* sites. The sizes of fragments derived from investigated plasmids differed from each other, suggesting that our library contains various *S. europaea* genes.

**FIGURE 2:**
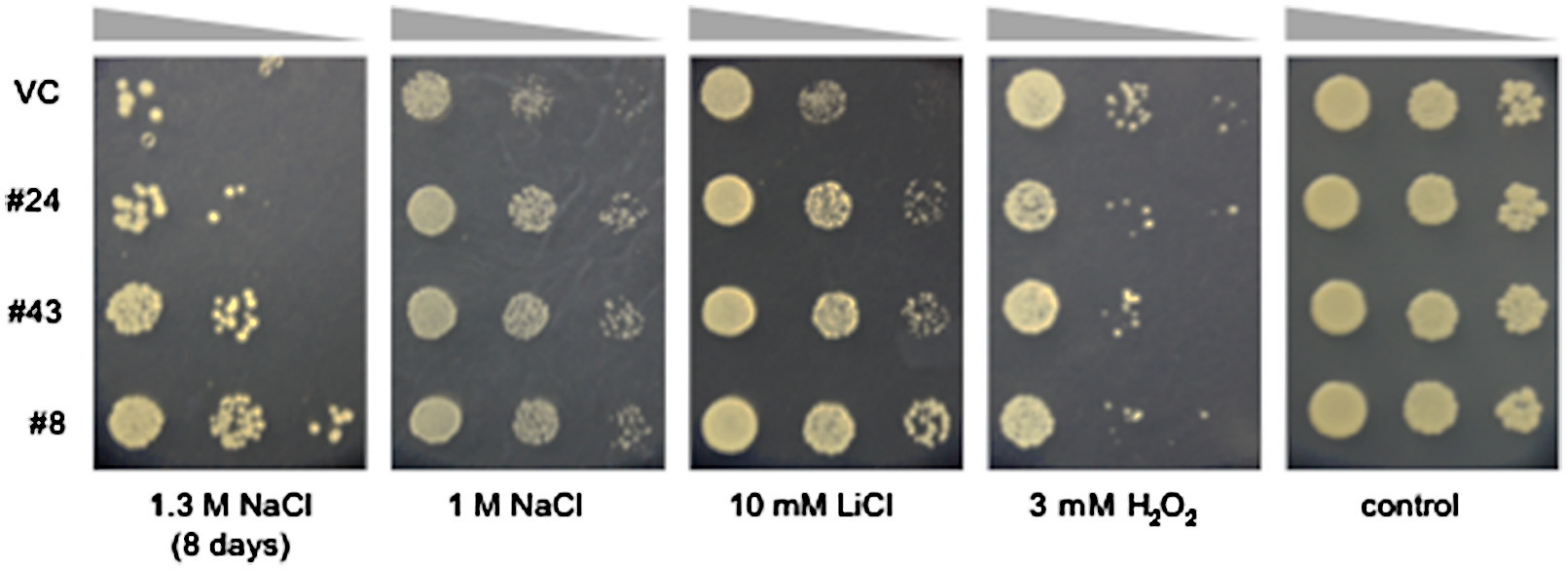
**Tolerance to NaCl, LiCl, and H_2_O_2_ in yeast lines #8, 24, and 43.** Overnight cultures (*OD*_600_ = 0.5) of the salt-tolerant yeast lines #8, 24, 43, and a vector control line (VC) were serially diluted and spotted onto media containing the indicated concentrations of NaCl, LiCl, or H_2_O_2_ and then grown for an additional 3 days, except for yeast cells grown on medium containing 1.3 M NaCl, which were grown for 8 days.

To screen *S. europaea* genes enhancing salt tolerance in eukaryotic cells, the cDNA library was introduced into *S. cerevisiae* BY4741 and salt-tolerant colonies were screened. Since wild-type yeast BY4741 shows salt sensitivity above from 500 mM NaCl, with complete growth arrest at 2.0 M NaCl (Eswaran et al., 2010), media containing 1.3 or 1.6 M NaCl were used for screening in the present study. The transformed yeasts were plated on media containing 1.3 M NaCl. Primary resistant colonies were harvested and re-plated on media containing 1.6 M NaCl. Three colonies, named #8, 24, and 43, were more tolerant to NaCl than a vector control strain (VC) transformed with pYES2.1/V5-His/lacZ. The plasmids harbored in these yeast lines were rescued and re-transformed into BY4741. The resulting transformants also showed improved tolerance to NaCl relative to the VC (**Figure 2**), suggesting the *S. europaea* genes carried by #8, #24, and #43 enhanced yeast salt tolerance. These genes were named *SeNN8*, *24*, and *43*, and their DNA sequences are reported in **Supplementary Figure S1**.

**FIGURE 3:**
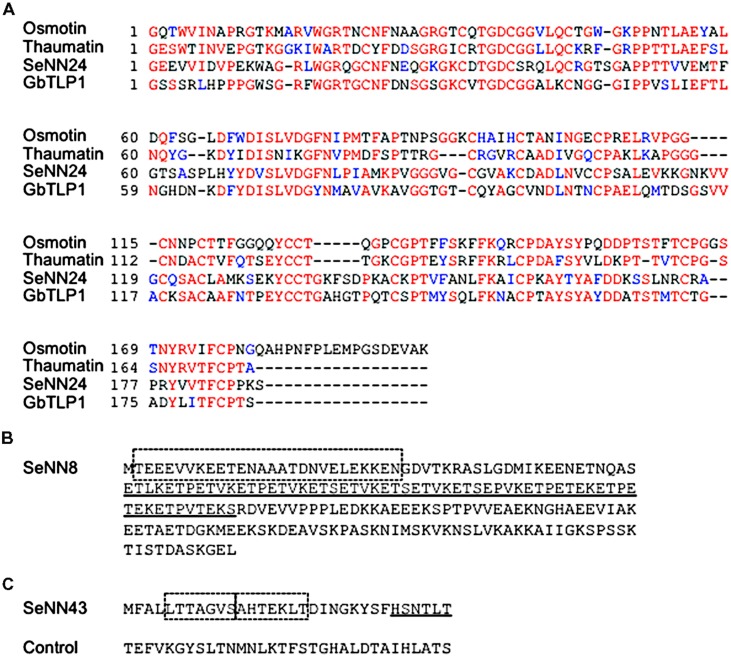
**Deduced amino acid sequences of SeNN24, 43, and 8. (A)** Alignment of deduced amino acid sequences of SeNN24 and various TLPs. The amino acid sequences of SeNN24, Osmotin (XP_009782398.1), Thaumatin (P02884.1), and GbTLP1 (ABL86687) were aligned using ClustalW (http: //www.genome.jp/tools/clustalw/) and then analyzed with BOXSHADE (http://www.ch.embnet.org/software/BOX_form.html). Red characters denote conserved invariant amino acids, and blue characters indicate conserved substitutions. **(B)** Deduced amino acid sequence of SeNN8. The dashed box indicates a predicted coiled-coil domain. The underline indicates a predicted low-complexity region. **(C)** Deduced amino acid sequence of SeNN43. The dashed boxes indicate predicted FHA domain-binding motifs. The underline indicates a predicted 14-3-3 protein-binding motif. The control peptide was used in **Figures 5, 6** and **8**.

Salinity causes hyperionic and hyperosmotic stresses. As a consequence of these primary effects, secondary stresses such as oxidative damage often occur. To determine whether the salt-tolerant phenotypes of the #8, #24, and #43 yeasts result from an improved tolerance to ionic, osmotic, or oxidative stresses, we examined growth of these yeasts on media containing an alkali ion (Li^+^) or a ROS (H_2_O_2_) (**Figure 2**). The three yeast lines and the VC showed no obvious differences in growth on media containing 3 mM H_2_O_2_, suggesting that *S. europaea* genes introduced in these lines are not involved in H_2_O_2_ scavenging. On the other hand, the three lines showed improved tolerance to 10 mM LiCl relative to the VC. Because of the vanishingly low osmotic pressure of the LiCl media, it is suggested that *SeNN8*, *24*, and *43* especially confer alkali ion tolerance to yeast. Alternatively, the Li^+^ tolerance and Na^+^ tolerance in these lines may not be due to same molecular mechanisms, since Li^+^ specifically exerts strong toxicity on media containing galactose as used in the present study (Masuda et al., 2001).

**FIGURE 4:**
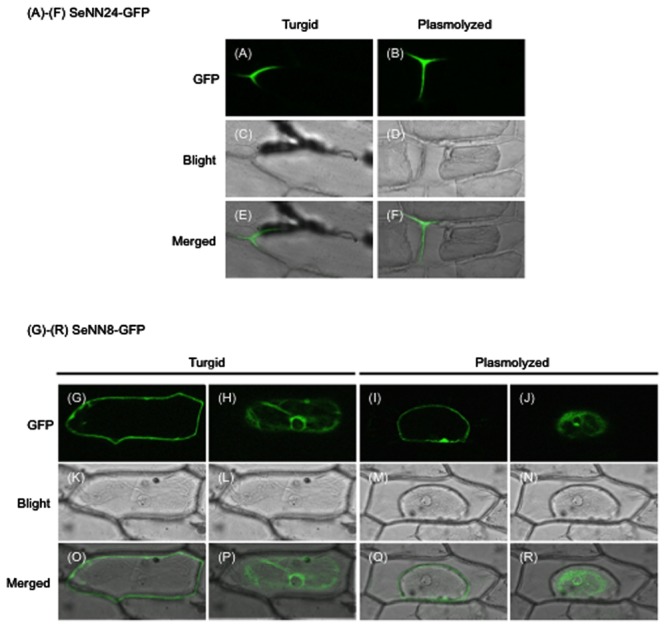
**Subcellular localization of SeNN24-GFP and SeNN8-GFP in onion epidermal cells. (A,B,G–J)** GFP fluorescence in onion cells transiently expressing SeNN24-GFP **(A,B)** or SeNN8-GFP **(G–J)**. The images were acquired before **(A,G,H)** or after **(B,I,J)** cell plasmolysis. The fluorescence images of SeNN8-GFP were acquired at two different depths in a same cell (**G,H** for a turgid cell, **I,J** for a plasmolyzed cell). **(C,D,K–N)** Bright-field images of the same cells as shown in the upper panels. **(E,F,O–R)** Bright-fields images merged with fluorescence images.

### Identification of a Gene Encoding a Thaumatin-like Protein

DNA sequencing revealed that the *SeNN24* mRNA has a 987-bp sequence with a 3′ poly(A) tail and that genomic *SeNN24* has an intron in its deduced open reading frame (**Supplementary Figure S1**). Additionally, there are two single-base differences between cDNA and genomic sequences of *SeNN24*. These differences may be due to single nucleotide polymorphisms among individuals used in each experiment. RT-PCR analysis revealed that *SeNN24* was expressed in *S. europaea* seedlings (**Supplementary Figure S2**).

The *SeNN24* mRNA was predicted to encode a protein composed of 248 amino acids similar to thaumatin-like proteins (TLPs) (**Figure 3A**). TLPs are the products of a large, highly complex gene family involved in host defense and a wide range of developmental processes in fungi, plants, and animals (Liu et al., 2010). Some TLPs respond to biotic and abiotic stresses in plants, and overexpression of TLPs such as osmotin and GbTLP1 confers resistance to fungal pathogens as well as tolerance to salinity and drought in transgenic plants (Munis et al., 2010; Subramanyam et al., 2012). The amino acid sequences of osmotin and GbTLP1 share 38 and 43% identity, respectively, with the deduced SeNN24 protein. The SeNN24 protein was predicted to be an extracellular protein by using SubLoc v1.0 program^1^, like GbTLP1.

To investigate the subcellular localization of SeNN24, a construct was produced in which SeNN24 was fused to GFP. The construct was transiently expressed in living onion epidermal cells, which were then subjected to analysis by confocal laser scanning microscopy. The fluorescence images showed a clear localization of GFP to the cell periphery (**Figures 4A–F**, **Supplementary Figures S3A**–**F**). For this experiment, we used both intact and plasmolyzed onion epidermal cells, which allowed us to differentiate between localization within the cell wall and within the plasma membrane. Our results indicate that SeNN24-GFP is localized to the cell wall.

### Identification of a Gene Encoding a Coiled-coil Protein

DNA sequencing revealed that the *SeNN8* mRNA is a 943-bp sequence with a 3′ poly (A) tail and that genomic *SeNN8* has no intron in its deduced open reading frame (**Supplementary Figure S1**). Additionally, there are three single-base differences between cDNA and genomic sequences of *SeNN8*. RT-PCR analysis revealed that *SeNN8* was expressed in *S. europaea* seedlings (**Supplementary Figure S2**).

There are several ATG codons in this sequence. If the first ATG is an initiation codon, the deduced amino acid sequence encoded by this gene consists of 211 amino acids (**Figure 3B**). The N-terminal region (amino acid position 2–27) was predicted to contain a short coiled-coil domain that likely mediates protein–protein interaction (SMART protein domain prediction^2^. The deduced SeNN8 protein is similar to C terminus of three proteins of *Beta vulgaris*, glutamic acid-rich protein (GARP)-like isoform X3, FK506-binding protein 5 (FKBP5)-like isoform X1, and X2 (BLAST^3^). The involvement of these in plant salt tolerance is not clear.

To investigate the subcellular localization of SeNN8, SeNN8-GFP fusion protein was transiently expressed in living onion epidermal cells, like the SeNN24-GFP fusion protein. The fluorescence images were acquired at two different depths (**Figures 4G,H,K,L,O,P**; **Supplementary Figures S3G,H,K,L,O,P**). Although the SeNN8 protein was predicted to be a nuclear protein by using SubLoc v1.0 program, the SeNN8-GFP fusion protein was localized to the nuclear periphery and the cell periphery in onion cells. In plasmolyzed cells, the SeNN8-GFP fusion protein was localized to the nuclear periphery and the plasma membrane (**Figures 4I,J,M,N,Q,R**; **Supplementary Figures S3I,J,M,N,Q,R**).

### Identification of a Gene Encoding a Novel Peptide of 32 Amino Acids

DNA sequencing revealed that the *SeNN43* mRNA has a 255-bp sequence with a 3′ poly(A) tail and that genomic *SeNN43* sequence is identical to that of cDNA (**Supplementary Figure S1**). RT-PCR analysis revealed that *SeNN43* was expressed in *S. europaea* seedlings (**Supplementary Figure S2**). There are three ATG codons in this sequence. If the first ATG is an initiation codon, the gene encodes a novel short peptide composed of 32 amino acids (**Figure 3C**). A novel class of genes encoding small peptides has been discovered in plant morphogenesis and responses to environmental stimuli (Fletcher et al., 1999; Dong et al., 2013; Bashir et al., 2014). These genes contain one or more short open reading frames of <100 codons and are translated into small peptides (Kastenmayer et al., 2006). The SeNN43 peptide was predicted to contain a 14-3-3 protein-binding motif, FHA domain-binding motifs, and phosphorylation sites, based on the ELM protein domain prediction program^4^.

**FIGURE 5:**
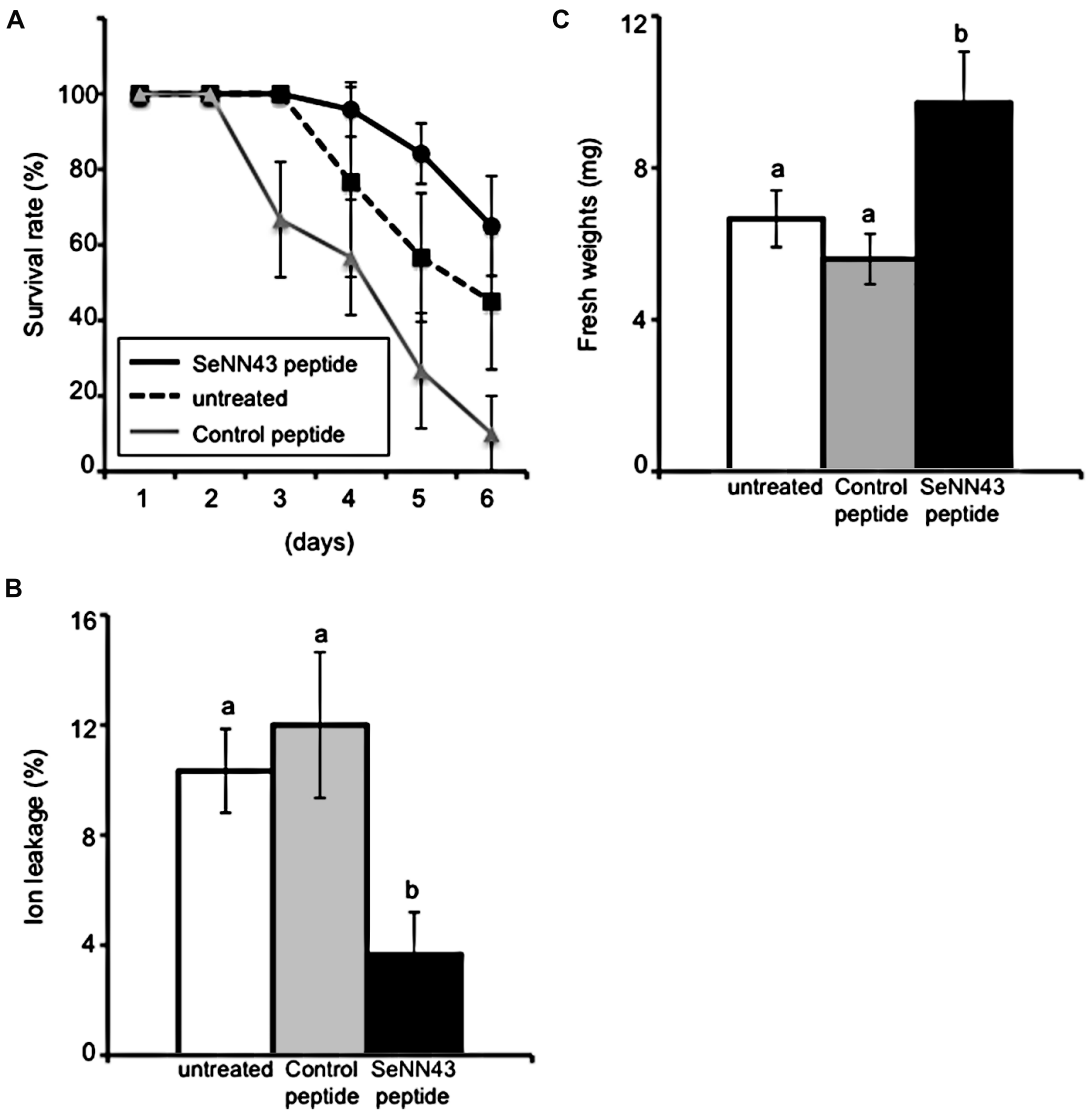
**Salt tolerance in *Arabidopsis* plants treated with the SeNN43 synthetic peptide. (A)** Survival of SeNN43-treated *Arabidopsis* plants on medium containing NaCl. Seven-day-old *Arabidopsis* plants were treated with 5 μM SeNN43 peptide (solid black line), control peptide (solid gray line), or water (dotted line) for 24 h, transferred to medium containing 200 mM NaCl, and then grown for an additional 6 days. The numbers of plants with chlorotic cotyledons were scored. **(B)** Ion leakage from leaves of the SeNN43-treated *Arabidopsis* plants on a medium containing NaCl. Seven-day-old *Arabidopsis* plants were treated with 5 μM SeNN43 peptide (black bar), control peptide (gray bar), or water (white bar) for 24 h, transferred to medium containing 200 mM NaCl, and grown for an additional 4 days. **(C)** Fresh weights of shoots of the SeNN43-treated *Arabidopsis* plants on medium containing NaCl. *Arabidopsis* plants were treated with peptides or water and subsequently with NaCl as shown in **(B)**. These plants were transferred to normal MS media and grown for an additional 3 days. Data are means ± SD of three replicates of 8–10 plants. Statistical significance was determined by Steel-Dwass test; significant differences (*P* < 0.05) are indicated by different lowercase letters.

In order to elucidate possible functions of this gene in plant salt tolerance, we examined the effects of exogenous application of a synthetic peptide of SeNN43 on salt tolerance in *Arabidopsis*. Roots of wild-type *Arabidopsis* seedlings were pretreated with the SeNN43 synthetic peptide, then incubated on media containing NaCl (see Materials and Methods). As a control, we used a peptide that has the same amino acid composition as SeNN43, but in a different order (**Figure 3C**). Survival of the SeNN43-treated seedlings was slightly but not significantly higher than those of the untreated and the control peptide-treated seedlings (**Figure 5A**).

Tissue damage in leaves was monitored by ion leakage, an indicator of plasma membrane damage. Pretreatment with the SeNN43 synthetic peptide alleviated tissue damage caused by NaCl (**Figure 5B**); in contrast, pretreatment with a control peptide did not alleviate tissue damage. These results suggest that the SeNN43 peptide enhances plant salt tolerance in a sequence-specific manner.

**FIGURE 6:**
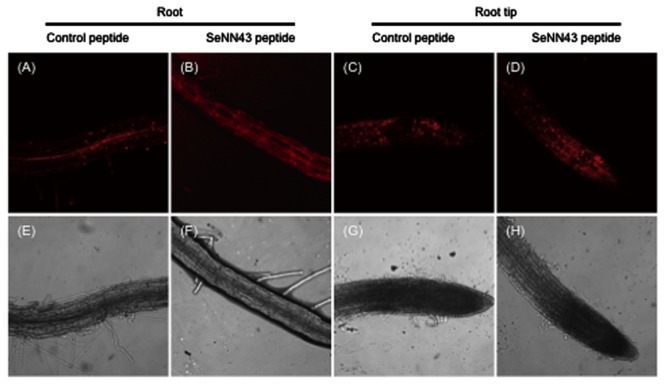
**Nuclear staining of non-viable cells in *Arabidopsis* roots under salt stress conditions.** Propidium iodide (PI)-stained cells in *Arabidopsis* roots **(A,B)** and root tips **(C,D)**. Seven-day-old *Arabidopsis* plants were treated with 5 μM control peptide **(A,C)** or SeNN43 peptide **(B,D)** for 24 h, transferred to medium containing 100 mM NaCl, and grown for an additional 4 days. The lower panels show bright-field images of the same cells as shown in the upper panels **(E–H)**.

**FIGURE 7:**
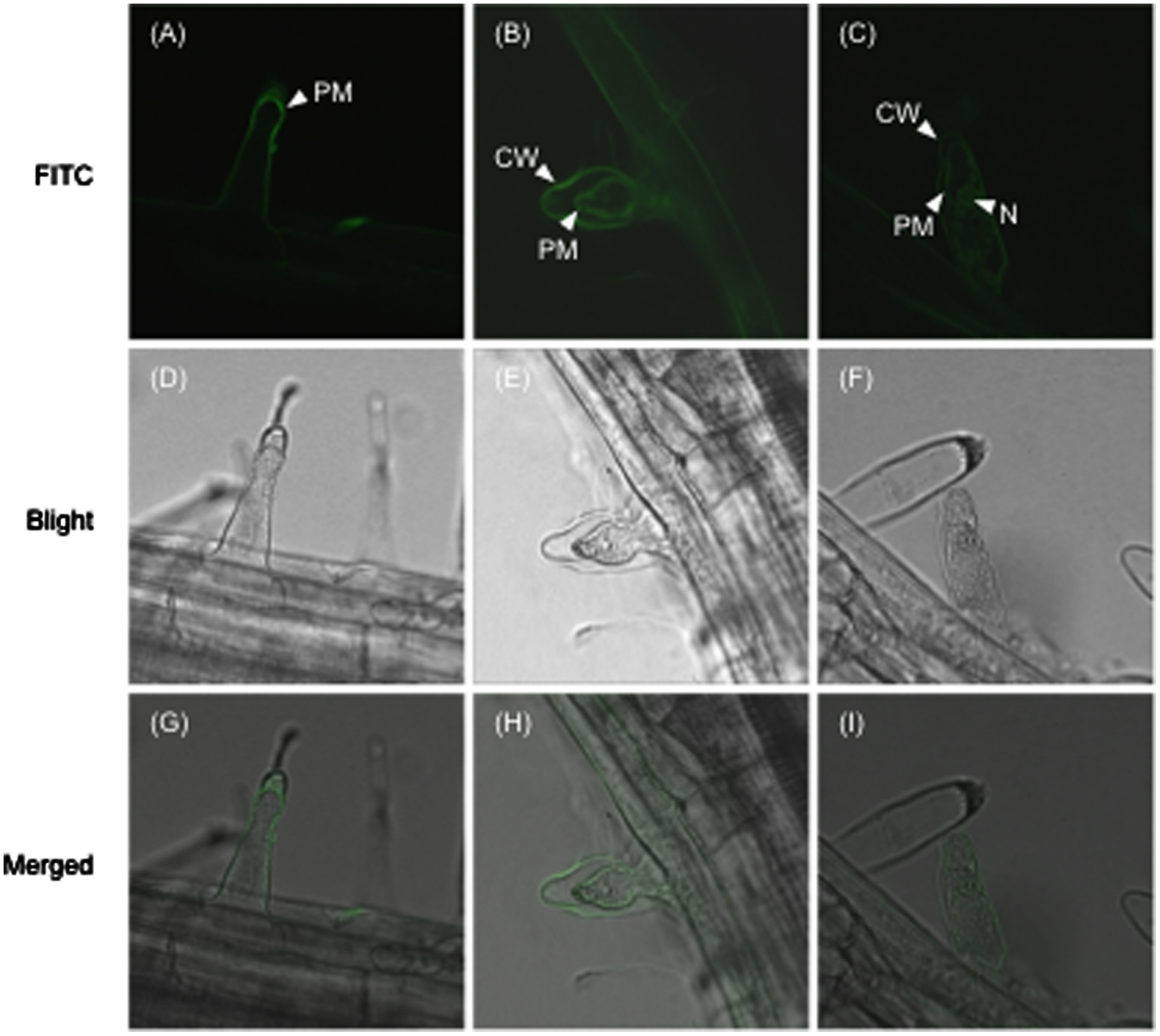
**Subcellular localization of the FITC-conjugated SeNN43 peptide in *Arabidopsis* root hairs. (A–C)** FITC fluorescence in *Arabidopsis* root hairs treated with the FITC-conjugated SeNN43 peptide. **(D–F)** Bright-field images of the same cells as shown in the upper panels. **(G–I)** Bright-fields images merged with fluorescence images. PM, CW, and N indicate the plasma membrane, the cell wall, and the nucleus, respectively.

Propidium iodide (PI), a DNA-binding red fluorescent dye, can only enter cells with damaged membranes. Bright PI fluorescence, particularly in nuclei, is an indicator of non-viable plant cells. As shown in **Figure 6A**, the nuclei of *Arabidopsis* root cells were strongly stained by PI after exposure to 100 mM NaCl and pretreatment with a control peptide, especially in stele cells. In contrast, pretreatment with the SeNN43 synthetic peptide reduced the number of PI-stained nuclei (**Figure 6B**) under the same conditions, suggesting the SeNN43 peptide enhances plant salt tolerance. In root tips, there were no obvious differences in the number of PI-stained nuclei between the SeNN43 peptide-pretreated and the control peptide-pretreated plants under NaCl conditions (**Figures 6C,D**). Thus, the SeNN43 peptide could not enhance the salt tolerance of root cells in meristematic and elongation zones. There were also no clear differences in root elongation rates under 100 mM NaCl conditions in either group: After 4 days NaCl treatment, 0.45 ± 0.19 cm (SeNN43) and 0.37 ± 0.17 cm (control), after 7 days NaCl treatment, 2.02 ± 0.10 cm (SeNN43), and 1.68 ± 0.13 cm (control), respectively.

In order to confirm the enhanced salt tolerance of SeNN43 peptide-pretreated plants, shoot fresh weights of *Arabidopsis* seedlings were measured after transient exposure to NaCl and after subsequent culture without NaCl. The fresh weights of the SeNN43 peptide-pretreated plants were significantly higher than those of the control peptide-pretreated and the untreated plants (**Figure 5C**).

To investigate the subcellular localization of SeNN43, a fluorescein isothiocyanate (FITC)-conjugated SeNN43 peptide was provided to *Arabidopsis* root and then FITC fluorescence was detected. In some cells, FITC fluorescence was localized to the plasma membrane, the cell wall, and the nucleus (**Figure 7**). Interestingly, strongly fluorescent cells showed retraction of the cytoplasm that is a characteristic hallmark feature of apoptosis-like programmed cell death (AL-PCD; Hogg et al., 2011).

**FIGURE 8:**
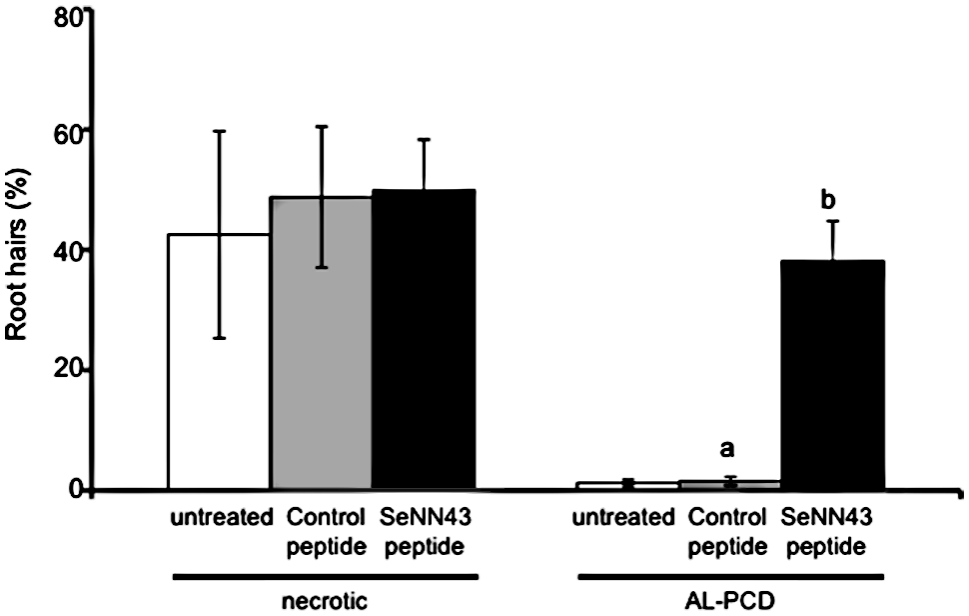
**Effects of the SeNN43 peptide on rates of AL-PCD in *Arabidopsis* root hairs.** Seven-day-old *Arabidopsis* plants were treated with 5 μM SeNN43 peptide (black bar), control peptide (gray bar), or water (white bar) for 24 h, and stained with FDA. Root hairs showing retraction and condensation of the cytoplasm and no FDA staining were deemed to have undergone AL-PCD. Those showing neither FDA staining nor retraction of the cytoplasm were scored as necrotic cells. Rates of AL-PCD or necrosis were plotted. Data are means ± SD of three replicates of 200–300 root hairs. Statistical significance was determined by student *t*-test; significant differences (*P* < 0.05) are indicated by different lowercase letters.

To investigate effects of the SeNN43 peptide on induction of AL-PCD, root hairs potentially undergoing AL-PCD were scored in untreated, the control peptide-treated, or the SeNN43 peptide-treated *Arabidopsis*. Roots after each treatment were stained with fluorescein diacetate (FDA), a fluorescent marker for living cells, and then three types of root hairs were scored: FDA-stained root hairs as living cells, no-stained root hairs with retraction of the cytoplasm as cells undergoing AL-PCD, and root hairs showing neither FDA staining nor retraction of the cytoplasm as necrotic cells (**Figure 8**). Using Steel-Dwass test for multiple comparisons, there were no significant differences in the percentages of both AL-PCD and necrosis among three treatment groups. However, using student *t*-test (*P* < 0.05) to compare the percentages of AL-PCD between the SeNN43-treated and the control peptide-treated plants, it was suggested that the SeNN43 peptide significantly induced AL-PCD in *Arabidopsis* root hairs.

## Discussion

### Three Genes of *S. europaea* were Identified by Functional Screening

To identify candidate genes encoding key proteins in salt tolerance in *S. europaea*, we constructed an *S. europaea* cDNA library. The plasmid libraries were transformed into *E. coli* and 233,760 independent clones were obtained. Recently, Ma et al. (2013) identified 109,712 unigenes from *S. europaea* through transcriptome analysis using next generation sequencing. Given PCR-amplification step in the construction of our *S. europaea* cDNA library, our library may not cover all genes in *S. europaea*. In our screening, three genes were identified as *S. europaea* genes enhancing yeast salt tolerance. Among them, only *SeNN24* encodes a protein homologous to a well-known protein involved in salt tolerance. In the present study, other previously reported *S. europaea* genes have not been screened, for example, *SeNHX1*, *SeCMO*, *SePSY*, and *SeLCY*, which can enhance plant salt tolerance (Han et al., 2008; Zhou et al., 2008; Wu et al., 2010; Chen et al., 2011; Zhang et al., 2014). This may result from the low number of independent clones in our library. Alternatively, it is possible that yeast does not have counterparts of *S. europaea* proteins which function coordinately with library proteins. For example, expression of an *Arabidopsis* plasma membrane Na^+^/H^+^ exchanger SOS1, a critical salt tolerance determinant in plants, slightly enhances salt tolerance in transgenic yeasts. To clearly enhance the yeast tolerance, co-expression of SOS2–SOS3 complex that activates SOS1 in plants is required (Quintero et al., 2011). This may be due to absence of SOS2–SOS3 counterpart in yeast. To overcome such difficulty, it is better that model plants are used as the host organism for functional screen of plant genes.

For our investigation of the functions of three genes identified in the present study, *Arabidopsis* overexpression lines and *S. europaea* knock-out lines will be generated. We have already succeeded in constructing transgenic *S. europaea* calli expressing GFP (data not shown).

### SeNN24

The *SeNN24* gene encodes a protein similar to thaumatin-like proteins (TLPs), polypeptides of about 200 amino acids that share sequence similarity with thaumatin, a sweet-tasting protein. TLPs have been discovered in a wide range of organisms, including plants (Liu et al., 2010). On account of their inducible expression by stresses such as pathogen attack, plant TLPs are classified as the pathogenesis-related protein family 5. Although many studies have demonstrated that constitutive expression of TLPs enhances salt and drought tolerance and fungal resistance in plants (Munis et al., 2010; Subramanyam et al., 2012), the molecular functions of TLPs in salt tolerance remain unknown. A sea-island cotton (*Gossypium barbadense* L.) TLP gene (*GbTLP1*) also enhances resistance against *Verticillium dahlia*, salinity and drought in transgenic tobacco (Munis et al., 2010). The GbTLP1 protein is predicted to be an extracellular protein and potentially involved in cotton fiber secondary cell wall development. Cell wall hardening is one of the main mechanisms of dehydration avoidance when plants are exposed to low water potential, such as drought and salinity (Verslues et al., 2006). The amino acid sequence of SeNN24 shares 43% identity with GbTLP1. Additionally, SeNN24 is thought to be an extracellular protein (**Figures 4A**–**F**; **Supplementary Figures S3A**–**F**). Therefore, SeNN24 may be also involved in cell wall development in *S. europaea*.

SeNN24 enhanced yeast tolerance to 10 mM LiCl (**Figure 2**). This may result from SeNN24-caused changes in composition of yeast cell wall. Lithium is very toxic to yeast cells growing in media with galactose as the sole carbon source. For production of glucose-6-phosphate (G6P), a starting material for glycolysis, there are galactose-dependent pathway and glcose-dependent pathway in yeast. Lithium specifically inhibits production of G6P via the former pathway (Masuda et al., 2001). In the present study, since we used media with galactose as the major carbon source for yeast growth test (precisely together with raffinose), glycolysis may be strongly inhibited by Li^+^ in the vector control yeast (**Figure 2**). It is known that several plant and fungal TLPs binds and hydrolyzes β-1,3-glucans, a major component of yeast cell wall (Liu et al., 2010). If SeNN24 also function as a β-1,3-glucanase, the SeNN24-expressing yeast can acquire glucose even on media without glucose as used in the present study by degradation of own cell wall. Incorporated glucose can be then converted to G6P via glcose-dependent pathway even in the existence of Li^+^. The increasing availability of glucose is an advantageous trait for Li^+^ tolerance on media with galactose as the major carbon source.

SeNN24 enhanced yeast tolerance to 1.3 M NaCl (**Figure 2**). Molecular mechanisms underlying the enhanced tolerance remain unclear. SeNN24-caused changes in cell wall may affect activities of osmotic sensor or Na^+^ transporter on the plasma membrane in yeast. In order to understand the physiological and molecular functions of *SeNN24*, further investigation is needed, such as a phenotypic analysis of *SeNN24*-overexpressing plants.

### SeNN8

There are several ATG codons in the *SeNN8* sequence. Among three ORFs, one could encode single amino acid, methionine, and another could encode a 26-amino-acids peptide. The latter peptide may be expressed in *S. europaea* and play a role in plant salt tolerance, like the SeNN43 peptide. In order to confirm the existence and function of the peptide, posttranslational analysis is needed. We will examine the effects of exogenous application of the synthetic 26-amino-acids peptide on salt tolerance in plants. The rest ORF could encode a 211-amino-acids protein with a coiled-coil domain that likely mediates protein–protein interactions, described above (**Figure 3B**).

The predicted coiled-coil domain in SeNN8 protein is very short (26 amino acids), similar to the TRK-T3 oncogene (Greco et al., 1995). Coiled-coil peptides consisting of 14 amino acids are able to form dimers (Dong and Hartgerink, 2006); thus, the SeNN8 protein may form homo- or heterooligomers via its coiled-coil domain. Preliminary results of a yeast two-hybrid assay indicated that the SeNN8 protein does not form a homooligomer (data not shown). In order to identify its interacting partners, we constructed an *S. europaea* cDNA library for a yeast two-hybrid screen. Identification of the interacting partners will provide valuable information regarding the precise roles of SeNN8.

The deduced SeNN8 protein is similar to C terminus of three proteins of *Beta vulgaris*, GARP-like isoform X3, FKBP5-like isoform X1, and X2. There are several reports about plant GARP-like proteins (Romo et al., 2002; de Souza et al., 2006). Although GARP-like proteins may be implicated in plant development, the involvement of these in plant salt tolerance is unknown. On the other hand, FKBP5 belongs to a family of immunophilins named for their ability to bind immunosuppressive drugs in human (Galat, 1993; Fruman et al., 1994; Jiang et al., 2008). This has peptidyl-prolyl isomerase activity, and also associates with chaperones to help protein folding. However, the possible functions of FKBP5 in plants remain unclear.

The similarity between SeNN8 and C terminus of three proteins raises the possibility that a full-length *SeNN8* may be longer than that isolated in the present study and the latter may be a 5′-terminal truncated artifact. However, we think that SeNN8 isolated in the present study is a full-length for the reason described below. Not mentioned above, we constructed two *S. europaea* cDNA libraries in the present study. These two libraries were derived from different individuals, independently extracted mRNAs and were constructed by using different cloning methods, each other. We performed yeast functional screening by using these two libraries, and isolated salt-tolerant colonies. Interestingly, exactly the same DNA sequence as SeNN8 was also isolated from both libraries. These 5′ and 3′ termini were also identical. Based on this result, *SeNN8* isolated in the present study is probably a full-length form.

The SeNN8 protein is predicted to be a nuclear protein with no transmembrane domain by using SubLoc v1.0 program and TMHMM program^5^. The calculated molecular weight of the SeNN8-GFP fusion protein is about 50 kDa. The size is sufficiently small to enter nuclei by passive diffusion. However, the SeNN8-GFP fusion protein was localized to the nuclear periphery and the plasma membrane in onion cells (**Figures 4G**–**R**; **Supplementary Figures S3G**–**R**). This raises the possibility that SeNN8 interacts with the plasma membrane-localized or the nuclear envelope-localized proteins of onion. SeNN8 may be involved in signal transduction of salt stress through interaction with or release from membrane-anchoring proteins in *S. europaea*.

### SeNN43

The *SeNN43* gene encodes a short peptide of 32 amino acids, including a predicted 14-3-3 protein-binding motif, FHA domain-binding motifs, and phosphorylation sites (ELM protein domain prediction program^4^). There are many genes encoding 14-3-3 proteins or FHA domain proteins in plants. These proteins interact with phosphorylated target proteins and function in signal transduction. For example, *Arabidopsis* 14-3-3 proteins AAA and κ interact with and suppress the activity of SOS2, a key kinase in the response to salt stress (Zhou et al., 2014). The FHA domain protein ABA1 functions in biosynthesis of the phytohormone abscisic acid, which plays critical roles in signal transduction in response to salt stress (Xiong et al., 2002).

Protein–protein interactions between 14-3-3 and target proteins are stabilized or inhibited by small molecules such as the 20-amino-acid peptide R18 (Milroy et al., 2013). In present study, exogenous application of the 32-amino-acid peptide SeNN43 enhanced salt stress tolerance in *Arabidopsis*. The SeNN43 peptide may interact with endogenous 14-3-3 proteins or FHA domain proteins to influence their activities, thereby improving salt tolerance.

Our result suggests that the SeNN43 peptide enhance the salt tolerance of root cells (**Figure 6**). Although the nuclei of control root cells were strongly stained by PI after exposure to NaCl, especially in stele, most of the nuclei of SeNN43-treated root cells were not satained except for meristematic and elongation zones. In meristematic and elongation zones, the nuclei of both SeNN43-treated and control root cells were equally damaged by NaCl. This may be a reason why there were no clear effects of the SeNN43 peptide on root elongation rates under salt stress conditions. These results suggest that the SeNN43 peptide specifically affects salt tolerance in differentiated tissues. The SeNN43 peptide may interact with 14-3-3 proteins or FHA domain proteins specifically expressed in differentiated tissues and regulate their activities.

Salinity induces apoptosis-like programmed cell death (AL-PCD) in roots of plants (Katsuhara, 1997; Huh et al., 2002). Although precise roles of salinity-induced PCD is unclear, it is suggested, as a hypothesis, that PCD-mediated elimination of root cells in response to salt shock is an adaptive mechanism that facilitates the production of roots more able to cope with a saline environment. The SeNN43 peptide induced cell death accompanied by retraction of the cytoplasm in *Arabidopsis* root hairs under none saline conditions (**Figure 8**), which is a characteristic hallmark feature of AL-PCD (Hogg et al., 2011). The SeNN43-induced cell death may result in enhancement of salt tolerance in the SeNN43-treated *Arabidopsis*. It is reported that 14-3-3 proteins are involved in sphingolipid-induced PCD in *Arabidopsis* (Lachaud et al., 2013). The SeNN43 peptide may interact with such 14-3-3 proteins and induce AL-PCD. To more precisely confirm the effect of SeNN43 on AL-PCD, we must examine other features of AL-PCD such as internucleosomal DNA cleavage and activation of caspase-like activities, in our future study.

Recent works reveal some peptides in plants are involved in intercellular signaling and stress adaptation, such as nitrogen deficiency (Tabata et al., 2014) and in induction of PCD (Blanvillain et al., 2011). Our results raise the possibility that the SeNN43 peptide, which can enhance plant salt tolerance, can be a novel member of such stress-related or signaling peptides.

Subcellular localization of SeNN43 was not clear in the present study, potentially because of exogenous application of the FITC-conjugated SeNN43 peptide (**Figure 7**). The physiological and molecular functions of *SeNN43* in *S. europaea* also remain unclear. Future analysis of the SeNN43 peptide, including a study of its protein–protein interactions, subcellular localization, effects on Na^+^ and Cl^-^ accumulation, and phenotypes of its overexpression lines will provide valuable information regarding the precise roles of the protein in salt tolerance of halophytes.

Exogenous application of the SeNN43 peptide enhanced plant salt tolerance, suggesting that agricultural use of the peptide may improve salt tolerance in crops without the need for transgenic approaches. Now we are trying to quantify effects of the SeNN43 peptide on salt tolerance in crops. This peptide may have a potential value as a novel agrochemical.

## Conflict of Interest Statement

The authors declare that the research was conducted in the absence of any commercial or financial relationships that could be construed as a potential conflict of interest.

## Footnotes

1 http://www.bioinfo.tsinghua.edu.cn/SubLoc/eu_predict.htm

2 http://smart.embl-heidelberg.de

3 http://blast.ncbi.nlm.nih.gov/Blast.cgi

4 http://elm.eu.org

5 http://www.cbs.dtu.dk/services/TMHMM/

## ABBREVIATIONS

SGR-Ura: synthetic complete media containing 2% galactose and 1% raffinose, and lacking uracil
VC: vector control
