# Supplementary material for: Yeast functional screen to identify genes conferring salt stress tolerance in Salicornia europaea
Source: Front Plant Sci. 2015 Oct 28;6:920. doi: 10.3389/fpls.2015.00920 (PMC4623525; doi:10.3389/fpls.2015.00920)
Supplement: Supplementary file 1 [file Data_Sheet_1.DOCX]

***SeNN24* cDNA**

atactcaccaaagttaagccATGATGAGGAATTTATTATTATTTTCCAGTCTCTTGTATCTTGCATTTTCTATGTTTTTAACAGATACTGATGCTGTTCAGCTAATCATCGTCAACAACTGTCACGAAGAAGTATGGCCCGCGATACTAGGAAACCCGGGGCAGCAGAGCCCTAAAGACGGTGGGTTCCACCTCAGCAGCGGTGAGGAAGTTGTTATAGACGTGCCTGAAAAATGGGCAGGAAGACTATGGGGTAGGCAAGGTTGCAACTTCAACGAACAAGGAAAAGGCAAATGTGACACAGGTGACTGTTCTAGACAACTGCAGTGCCGCGGCACCAGTGGGGCCCCTCCTACAACGGTGGTGGAAATGACATTTGGGACCTCCGCCTCCCCGCTGCATTACTATGATGTCAGCCTGGTGGACGGATTCAACTTGCCCATTGCTATGAAGCCTGTAGGAGGTGGGGTCGGATGTGGGGTCGCTAAATGTGATGCAGATCTTAATGTGTGTTGCCCATCAGCATTGGAAGTGAAGAAGGGAAACAAAGTGGTTGGGTGTCAAAGTGCTTGTTTGGCTATGAAATCTGAAAAATACTGCTGTACAGGAAAGTTTTCAGATCCTAAAGCATGCAAGCCAACAGTCTTTGCTAATCTGTTCAAAGCTATTTGTCCAAAGGCTTACACTTATGCCTTTGATGATAAGTCTAGTCTTAACAGATGCAGAGCTCCTAGATATGTCGTAACATTCTGCCCTCCTAAGTCCTAAgtagttgttctaagaaataagcttaattatgccctcctaagtcctaagcagattatcctgtactatgtaatttcaaaaagctctttttaaatttncagtgtttcagaagagaatagttcagtgaacatttgcatactgccttgggcaatacaagcagattcaacaacaagatttgaatgcaaactgtttaaatgactaaacagaatcctcattttcTGAGAAAAAAAAAAAAAAAAAAA

***SeNN24* genomic DNA**

ATGATGAGGAATTTATTATTATTTTCCAGTCTCTTGTATCTTGCATTTTCTATGTTTTTAacaggtattactgctttctttacttcaacatgtttgattttgcaagaaacatatagaacaactaacttaaacactgtttttctgttcacttacagATACTGATGCTGTTCAGCTAATCATCGTCAACAACTGTCACGAAGAAGTATGGCCCGCGATACTAGGAAACCCGGGGCAGCAGAGCCCTAAAGACGGTGGGTTCCACCTCAGCAGCGGTGAGGAAGTTGTTATAGACGTGCCTGAAAAATGGGCAGGAAGACTATGGGGTAGGCAAGGTTGCAACTTCAACGAACAAGGAAAAGGCAAATGTGACACAGGTGACTGTTCTAGACAACTGCAGTGCCGCGGCACCAGTGGGGCCCCTCCTACAACGGTGGTGGAAATGACATTTGGGACCTCCGCCTCCCCGCTGCATTACTATGATGTCAGCCTGGTGGACGGATTCAACTTGCCCATTGCTATGAAGCCTGTAGGAGGTGGGGTCGGATGTGGGGTCGCTAAATGTGATGCAGATCTTAATGTGTGTTGCCCATCAGCATTGGAAGTGAAGAAGGGAAACAAAGTGGTTGGGTGTCAAAGTGCTTGTTTGGCTATGAAATCTGAAAAATACTGCTGTACAGGAAAGTTTTCAGATCCTAAAGCATGCAAGCCAACAGTCTTTGCTAATCTGTTCAAGGCTATTTGTCCAAAGGCTTACACTTATGCCTTTGATGATAAGTCTAGTCTTAACAGATGCAGAGCTCCTAGATATGTTGTAACATTCTGCCCTCCTAAGTCC

***SeNN8* cDNA**

AGATGACAGAGGAAGAAGTGGTTAAGGAAGAGACAGAGAATGCTGCAGCAACTGATAATGTTGAGTTGGAAAAGAAGGAAAATGGTGATGTTACTAAAAGAGCAAGTCTTGGAGACATGATTAAGGAGGAAAATGAGACCAATCAAGCTTCAGAAACTCTGAAAGAAACACCAGAAACTGTGAAAGAAACACCTGAAACTGTTAAAGAAACATCAGAAACTGTTAAAGAAACGTCAGAAACTGTTAAAGAAACATCAGAACCTGTGAAAGAAACACCAGAAACTGAGAAAGAAACACCAGAAACTGAGAAAGAAACACCAGTAACTGAGAAAAGCAGAGATGTTGAAGTTGTTCCTCCTCCTTTGGAAGACAAGAAAGCAGAAGAAGAGAAATCTCCGACTCCCGTTGTAGAAGCAGAGAAAAATGGACATGCTGAGGAAGTTATTGCCAAGGAAGAAACAGCAGAAACTGATGGAAAGATGGAAGAGAAAAGCAAGGATGAAGCTGTAAGCAAGCCTGCTTCAAAGAATATCATGTCAAAGGTGAAGAACTCTCTAGTGAAAGCGAAGAAGGCGATTATTGGGAAATCTCCTTCCTCAAAGACGATCTCAACTGATGCTAGTAAGGGAGAATTGTAAGTTTGTATTTATGTATTTTGAAGGTGGTTTTCTTGTGAAACTGTGTTTTTCTGTATCATATTTTTGGTGTTTGTAATGTGGTTGTGGTTGTGTTTGATGGCTTGTTTTGGTTGATTTTTTTTTTATTATTTTTTATTTATTTTTTGTATTGTGTTTCATAGAATGTGAATACACAGAAGAGTATAGGTTTGAGGTTCTGTGAACTTCAGGATTTCATTGGTTTTTGTTTTGTTGTTTCATTGAAATTCTTATTAGTAATGTAATTTGTTCAAGCTTATGAATCAAGTAAATTGTATTTGTTTGCTAAAAAAAAAAAAAAAAAAAAAAAAAAAAA

***SeNN8* genomic DNA**

ATGACAGAGGAAGAAGTGGTTAAGGAAGAGACAGAGAATGCTGCAGCAACTGATAATGTTGAGTTGGAAAAGAAGGAAAATGGTGATGTTACTAAAAGAGCAAGTCTTGGAGACATGATTAAGGAGGAAAATGAGACCAATCAAGCTTCAGAAACTCTGAAAGAAACACCAGAAACTGTGAAAGAAACACCTGAAACTGTTAAAGAAACATCAGAAACTGTTAAAGAAACATCAGAAACTGTTAAAGAAACATCAGAACCTGTGAAAGAAACACCAGAAACTGAGAAAGAAACACCAGAAACTGAGAAAGAAACACCAGTAACTGAGAAAAGCAGAGATGTTGAAGTTGTTCCTCCTCCTTTGGAAGACAAGAAAGCAGAAGAAGAGAAATCTTCGACTCCCGTTGTAGAAGCAGAGAAAAATGGACATGCTGAGGAAGTTATTGCCAAGGAAGAAACAGCAGAAACTGATGGAAAGATGGAAGAGAAAAGCAAGGATGAAGCTGTAAGCAAGCCTGCTTCAAAGAATATCATGTCAAAGGTGAAGAACTCTCTAGTGAAAGCGAAGAAGGCGATTATTGGGAAATCTCCTTCCTCAAAGACAATCTCAACTGATGCTAGTAAGGGAGAATTG

***SeNN43* cDNA**

TTCTTTTCTTTACTTTTCTTTTTTTTTTTTTTTTTTTTTTTTCTGTACATTGCTTTTCTAAAGAAAAGAATAAATGTTTGCTCTGCTGACGACTGCTGGTGTTTCTGCTCATACTGAGAAGTTGACTGATATCAATGGGAAATACTCCTTTCATTCTAATACTCTCACTTGAGTATTCTTTGTAAATTGTAACCCTCTAGTTGAGTTTTAATTTCATGGGGGTGTAATAAAATCTAAATTAACATAATTGACATGAAAAAAAAAAAAAAAAAAAAAAAAAAAAAAAAAAAAAAAAAAAAAAAAAAAAAAAAAAAAAAAAAAAAAAAAAAAAAAAAAAAAAAAA

***SeNN43* genomic DNA**

ATGTTTGCTCTGCTGACGACTGCTGGTGTTTCTGCTCATACTGAGAAGTTGACTGATATCAATGGGAAATACTCCTTTCATTCTAATACTCTCACT

**Figure S1.** DNA sequences of *SeNN8*, *24*, and *43*.

Red characters indicate predicted start and stop codons. Underlined and large characters indicate single nucleotide differences between cDNA and genomic DNA sequenced in the present study. Lowercases indicate a possible intron in the *SeNN24* open reading frame. The DNA sequence data for *SeNN8*, *24*, and *43* have been deposited into the DDBJ database (accession numbers LC089023, LC089021 and LC089025 for cDNA, LC089024, LC089022 and LC089026 for genomic DNA, respectively).
