# Supplementary material for: Yeast functional screen to identify genes conferring salt stress tolerance in Salicornia europaea
Source: Front Plant Sci. 2015 Oct 28;6:920. doi: 10.3389/fpls.2015.00920 (PMC4623525; doi:10.3389/fpls.2015.00920)
Supplement: Supplementary file 2 [file Data_Sheet_2.PDF]

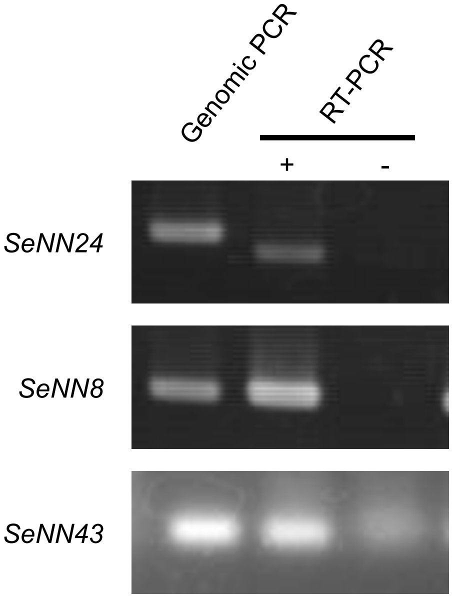

Figure S2. Genomic PCR and RT-PCR analysis of *SeNN8*, *24*, and *43*. Genomic DNA and total RNA isolated from *S. europaea* were used for the analysis. In RT-PCR analysis, reversetranscription solutions (+) or negative control solutions without reversetranscriptase (-) were used as PCR templates.
