# Supplementary material for: Yeast functional screen to identify genes conferring salt stress tolerance in Salicornia europaea
Source: Front Plant Sci. 2015 Oct 28;6:920. doi: 10.3389/fpls.2015.00920 (PMC4623525; doi:10.3389/fpls.2015.00920)
Supplement: Supplementary file 3 [file Data_Sheet_3.PDF]

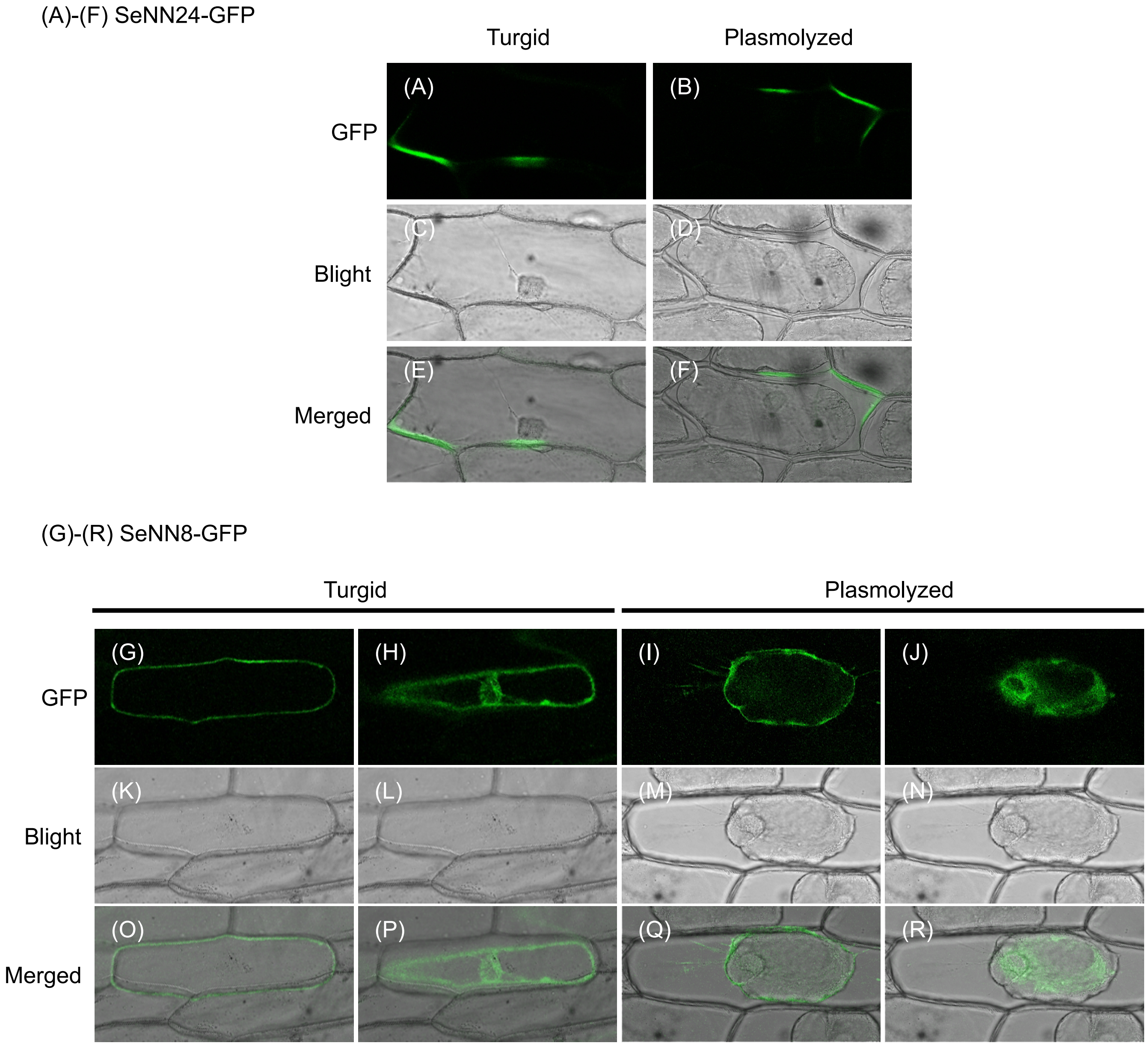

Figure S3. Reproducibility of subcellular localization analysis of SeNN24-GFP and SeNN8-GFP in Figure 4.
(A, B and G-J) GFP fluorescence in onion cells transiently expressing SeNN24-GFP (A and B) or SeNN8-GFP (G-J). The images were acquired before (A, G and H) or after (B, I and J) cell plasmolysis. The fluorescence images of SeNN8-GFP were acquired at two different depths in a same cell (G and H for a turgid cell, I and J for a plasmolyzed cell). (C, D and K-N) Bright-field images of the same cells as shown in the upper panels. (E, F and O-R) Bright-fields images merged with fluorescence images.
